# Prioritization of chemicals in food for risk assessment by integrating exposure estimates and new approach methodologies: A next generation risk assessment case study 

**DOI:** 10.3389/ftox.2022.933197

**Published:** 2022-09-19

**Authors:** Mirjam Luijten, R. Corinne Sprong, Emiel Rorije, Leo T. M. van der Ven

**Affiliations:** ^1^ Centre for Health Protection, Bilthoven, Netherlands; ^2^ Centre for Nutrition, Prevention and Health Services, Bilthoven, Netherlands; ^3^ Centre for Safety of Substances and Products, National Institute for Public Health and the Environment (RIVM), Bilthoven, Netherlands

**Keywords:** high-throughput screening, Monte Carlo Risk Assessment tool, new approach methodologies, next generation risk assessment, dietary exposure, threshold of toxicological concern, toxicity prediction models

## Abstract

Next generation risk assessment is defined as a knowledge-driven system that allows for cost-efficient assessment of human health risk related to chemical exposure, without animal experimentation. One of the key features of next generation risk assessment is to facilitate prioritization of chemical substances that need a more extensive toxicological evaluation, in order to address the need to assess an increasing number of substances. In this case study focusing on chemicals in food, we explored how exposure data combined with the Threshold of Toxicological Concern (TTC) concept could be used to prioritize chemicals, both for existing substances and new substances entering the market. Using a database of existing chemicals relevant for dietary exposure we calculated exposure estimates, followed by application of the TTC concept to identify substances of higher concern. Subsequently, a selected set of these priority substances was screened for toxicological potential using high-throughput screening (HTS) approaches. Remarkably, this approach resulted in alerts for a selection of substances that are already on the market and represent relevant exposure in consumers. Taken together, the case study provides proof-of-principle for the approach taken to identify substances of concern, and this approach can therefore be considered a supportive element to a next generation risk assessment strategy.

## Introduction

In recent years, various efforts have been made worldwide to contribute to innovating risk assessment of chemical substances, including design of new concepts building on integration of exposure and hazard assessment models and implementation of new approach methodologies (NAMs). A key element in here is the prediction of internal substance exposure concentrations in humans and extrapolation of *in vitro* or *in silico* hazard estimations to the human situation (National Research Council 2007, 2012; Embry et al., 2014; Pastoor et al., 2014; Berggren et al., 2017; Ball et al., 2022; Pallocca et al., 2022). The common goal is to establish a knowledge-driven system that allows for cost-efficient assessment of human health risks related to chemical exposures, without additional use of experimental animals. Generally, next generation risk assessment is considered feasible given the advances made in science and technology, exemplified by the development of models for rapid exposure predictions (Biryol et al., 2017; Thomas et al., 2019), high-throughput toxicokinetic modeling (Wambaugh et al., 2015; Pearce et al., 2017; Wambaugh et al., 2018; Wambaugh et al., 2019; Beal et al., 2022; Williams et al., 2022), and innovative (high-throughput) *in vitro* approaches for toxicity testing (Judson et al., 2010; Padilla et al., 2012; Richard et al., 2016; Loo and Zink 2017; Zurlinden et al., 2020; Ciallella et al., 2022; Klutzny et al., 2022). At the same time, it has been highlighted that the increasing number of chemicals poses a problem that cannot be solely addressed by next generation risk assessment, because of the complexity of the interactions between chemicals and biological systems (Fenner and Scheringer 2021).

Practical implementation of the concepts for next generation risk assessment has proven to be challenging, because it requires integration and assembly of elementary data with high levels of uncertainty into robust predictions for human risk. Explorations to this aim are accomplished in case studies (Judson et al., 2015; Silva et al., 2015; Doe et al., 2016; Wolf et al., 2016; De Abrew et al., 2019; Turley et al., 2019; Dent et al., 2021; Gilmour et al., 2022). Case studies can be helpful, as they demonstrate how tools and methods may be used to generate the information necessary for chemical risk assessment, and reveal possibilities and limitations to guide further development of methods and protocols. Additionally, they allow for comparison of conclusions with those of traditional approaches to risk assessment. In a previous case study we explored how *in silico* and high-throughput screening (HTS) approaches may be used for predicting toxicological effects in humans (van der Ven et al., 2020). In the present case study we build on that initial strategy for predicting toxicological hazard, and aim to pinpoint how to select substances to be subjected to HTS from the vast and ever growing volume of existing and newly developed substances. For that purpose of prioritization of chemical substances to undergo hazard assessment, we choose to apply an exposure-driven approach, combined with *in silico* methods to estimate toxicological activity of selected substances. Prioritization is aimed at identifying substances that may be of higher concern and is not intended as replacement of the actual risk assessment. Substances with higher priority do not necessarily pose a higher health risk than substances of lower priority.

Frequently, exposure assessment is conducted using tiered approaches to limit resources. Such a tiered approach usually starts with simple deterministic, but conservative, models requiring limited data input, followed by more sophisticated probabilistic models, which can therefore be more realistic. In its guidance on harmonized methodologies for human health, animal health and ecological risk assessment of combined exposure to multiple chemicals, EFSA provided a nice example of such a tiered approach in exposure assessment (European Food Safety Authority 2019a); this example is summarized in Table 1. It should be noted that the proposed tiering for data input, i.e., concentration data and consumption data, is flexible rather than fixed, depending on the data available. Consequently, concentration data and consumption data do not necessarily match in terms of tiers used for a risk assessment. For example, exposure to pesticides could be estimated using a combination of monitoring data (tier two concentration data) and individual consumption data (tier three consumption data), as has been done by EFSA in the risk assessment of combined exposure to pesticides relevant for the thyroid, the nervous system and acetylcholinesterase inhibition (European Food Safety Authority et al., 2021). Alternative methods to estimate exposure, particularly useful for substances that have not yet been marketed, include (high-throughput) models such as the Stochastic Human Exposure and Dose Simulation (SHEDS) model form U.S. EPA, field trial studies, and exposure modeling based on known fate in the field of existing contaminants.

**TABLE 1:** Example of tiering in exposure assessment^a^.

Tier	Concentration data	Consumption data	Exposure estimate
0	Permitted levels	Portion sizes	Semi-quantitative, Point estimates
1	Modelled and experimental data	Food balance sheet, Food baskets	Deterministic
2	Monitoring surveys	Summary statistics	Semi-probabilistic
3	Individual co-occurrence data	Individual data	Probabilistic

a Example taken from EFSA (European Food Safety Authority 2019a).

Key questions in the present case study were how to use exposure data for prioritization of substances within large databases of potential contaminants as explained above, and whether such an approach is feasible for new substances entering the market. For that purpose, we applied toxicological hazard estimation as the second identifier in our approach. EFSA in their guidance on grouping chemical into assessment groups for cumulative risk assessment proposed three prioritization methods (European Food Safety Authority et al., 2021). While two methods are specifically equipped for establishing mixtures, the risk-based prioritization which uses the hazard quotient (HQ; exposure divided by the health-based guidance value) is particularly useful for single chemicals as intended in our study. However, as this requires sufficient hazard data to derive health-based guidance values, this methodology is less feasible for new substances entering the market. For those substances, the Threshold of Toxicological Concern (TTC) concept, which is an estimation of toxicological activity based on chemical structure similarities, could be used.

To test our prioritization approach, we focused on chemicals in food, using a database of existing chemicals relevant for dietary exposure that comprises particularly pesticide residues, environmental pollutants and mycotoxins. This database was used to calculate exposure estimates, followed by application of the TTC concept. Substances were considered of higher priority in case exposure estimates exceeded the threshold (HQ > 1), or in case substances belong to exclusionary categories of the TTC concept (e.g., metals). This selection marks the final prioritization result. The thus identified ‘priority substances’ should be tested for toxicological potential in a follow-up assessment, e.g., through using HTS prediction approaches and subsequent testing in more complex test systems (Luijten et al., 2020; van der Ven et al., 2020). To illustrate how this could work out, we checked toxicological potency of the top priority substances in a selection of such HTS models. The findings for the case study are described and the applicability of this approach for new substances is discussed.

## Materials and methods

### Substances with exposure data

The case study presented here employed an exposure-driven approach, with a focus on chemicals in food. While recognizing the obvious limitation of this approach (see Discussion section for details), substances with available exposure data were identified using relevant monitoring data submitted to EFSA (European Food Safety Authority) in EFSA’s Standard Sample Description one format (SSD1; (European Food Safety Authority, 2010). For this, a database developed in the Horizon2020 project “EuroMix” was used (Crepet et al., 2019). This “EuroMix database” contains data on pesticide and contaminant concentrations in food items from 2010 to 2014 from eight European countries (Tier two concentration data, Table 1). From these data, chemicals in the SSD1 concentration database coded with substance codes with the extension “PPP” (pesticides), “ORG” (organic contaminants), “TOX” (toxins), “CHE” (other chemicals in food, such as metals and nitrates) or “PAR” (miscellaneous chemicals) were selected.

### Estimation of dietary exposures

For food consumption, data for the Dutch population aged 7–69 years were used (Van Rossum et al., 2011) and coded according to EFSA’s FoodEx1 system (European Food Safety Authority, 2011). This dataset contains the dietary habits of 3,819 Dutch subjects collected on two non-consecutive days over the period 2007 to 2010. Dietary habits were recorded using the 24-h recall method or, in case of young children, via the dietary record method. Results of the consumption survey were weighted for small deviances in sociodemographic characteristics in order to give results that are representative for the Dutch population (Tier three consumption data, Table 1). Chemicals are often measured in raw agricultural products rather than food products as consumed. Therefore, to translate concentrations in raw agricultural products into concentrations in foods as consumed, we used the Dutch food translation table, which describes consumed foods as their corresponding percentages of raw agricultural commodities (Boon et al., 2015); e.g., the % of raw, unprocessed, wheat in bread). Further refinement of final concentrations in consumed foods could be achieved through application of processing factors to correct for food processing leading to changes in substance concentrations (e.g., milling of wheat and or baking of bread). Such processing factors were however not always present or coded according to commonly used formats and therefore not applied. Although this may lead to an under- or overestimation of exposure, the current approach was considered appropriate for the purpose of this case study, namely prioritization of substances.

Calculations of exposure estimates for the substances of interest (Supplementary Table S1) were performed using the Monte Carlo Risk Assessment tool (MCRA; version 8.2; https://mcra.rivm.nl; Tier two exposure estimate, Table 1). Ideally, exposure estimates are calculated with a tool that automatically generates exposure estimates for all substances present in a concentration database, since single exposure assessment of large numbers of substances is time consuming and costly. At the time of conducting the analyses this feature was not yet integrated in MCRA, but the cumulative dietary exposure assessment module in MCRA could be used as a work-around. By using a relative potency of one for all substances, exposure estimates can be obtained for randomly compiled subsets of a maximum of 200 substances. This work-around can lead to slightly different exposure percentiles per substance than achieved with single substance MCRA modeling. The produced exposure estimates are therefore suitable for the present case study, but would require additional review before using them as actual exposures in the Netherlands.

As the TTC concept is only applicable for chronic exposure, we performed chronic exposure assessments, using the observed individual mean (OIM) model which calculates the intake per day for each subject and averages the intake of the two recorded days per subject (MCRA Reference Manual; mcra.rivm.nl). Exposure calculations were performed for upper and lower bound scenarios. In brief, in the lower bound scenario it is assumed that substances in foods below the level of detection (LOD) or level of quantification (LOQ) are absent, while in the upper bound scenario non-detects at the level of detection (LOD) or level of quantification (LOQ) are assumed to equal the value of the LOD or LOQ. For this study, the upper bound approach was preferred in view of the considerable reduction of the number of entries (i.e., (groups of) chemicals or residues thereof) when assigning representative structures, i.e., from 1,057 to 517 entries (see Results).

### Threshold of toxicological concern

The TTC concept was applied to the substances for which exposure estimates were calculated. To this end different software packages were used to evaluate the threshold applicable to each substance. ToxTree was used to assign Cramer classes to the substances (see step 3 below), the OECD QSAR Toolbox and DEREK Nexus were used to evaluate the potential mutagenicity of substances (step 2 below), and Accord for Excel was used in the third step of the evaluation. These software tools all require a chemical structure; therefore only those entries with a representative structure coupled to an SSID1 code in the EuroMix database were included in our evaluation. For these substances, the TTC was derived using the following approach: 1) evaluation of the TTC exclusion criteria; 2) determination of the presence of alerts for genotoxicity; 3) determination of the presence of organophosphate or carbamate functionality in the chemical structure; and 4) determination of the Cramer class of toxicity. Each step is discussed in detail below.

Regarding the evaluation of the exclusion criteria for the TTC concept (step 1) the list of substances with exposure estimates was screened manually to determine whether any exclusion rule applied. Substances that are inorganic, metal containing, mixtures, bioaccumulating, or aflatoxin-like were excluded from the TTC approach as no safe level of exposure can be determined for such substances (European Food Safety Authority and World Health Organization, 2016; European Food Safety Authority, 2019b). Substances for which other exclusion criteria apply, like proteins, nanomaterials and steroids, were not present in the list. For substances excluded from the TTC concept no safe threshold for exposure can be assigned. In principle any exposure of such substances gives rise to concern, and hence follow-up for these substances is required to estimate their potential risk.

Regarding step 2, the evaluation of the genotoxic potential of the substances, both the OECD QSAR Toolbox (version 4.3, http://www.oecd.org/chemicalsafety/risk-assessment/theoecdqsartoolbox.htm) and Derek Nexus (v2.2, knowledgebase 2018) software were applied to screen for the presence of alerts for genotoxicity in the chemical structures. Alerts from three modules in the DEREK Nexus software were taken as indicators of genotoxic potential: mutagenicity *in vitro* bacterium, chromosome damage *in vitro* mammalians, and chromosome damage *in vivo* mammalians. From the OECD QSAR Toolbox four endpoint specific profiles were considered as indicators of genotoxic potential: two profiles provided by the Instituto Superiore de Sanita (ISS) in Rome, Italy named “*in vitro* mutagenicity (Ames) alerts by ISS” and “*in vivo* mutagenicity (Micronucleus) alerts by ISS” and two profiles provided by LMC University of Bourgas, Bulgaria named “DNA alerts for AMES by OASIS” and “DNA alerts for CA and MNT by OASIS.” Alerts for bacterial mutagenicity (Ames test, point mutations in bacterial DNA) and chromosome damage (*in vitro* and/or *in vivo*) were evaluated separately according to the rule that two of the three models (DEREK, ISS or OASIS) for either Ames mutagenicity, or chromosome damage should be positive (identify at least one substructure alert). Overall, a substance was considered genotoxic if the evaluation for Ames mutagenicity OR chromosome damage was positive. Substances evaluated in this way as (potentially) genotoxic were subsequently assigned the TTC of 0.0025 μg/kg body weight/day.

Step 3, i.e., evaluation whether a representative chemical structure contains an organophosphate or carbamate functionality, was performed using the Accord for Excel add-in (BioVia, https://www.3dsbiovia.com/products/datasheets/accord-for-excel.pdf) and filtering the table of chemical structures for substructures phosphate (O=P (-O-R1) (-O-R2)-O-R3 or carbamate R1-O-C (=O)-N (-R1)-R2, with R1, R2, and R3 defined as any non-hydrogen substituent). Substances that were not assigned the TTC for genotoxic substances (step 2), but did contain organophosphate or carbamate functionalities were assigned a TTC of 0.3 μg/kg body weight/day.

Regarding step 4, the Toxtree software (v3.1, http://toxtree .sourceforge.net) was applied using the decision tree “Cramer Classes, with extensions” in order to assign a Cramer class (Cramer et al., 1978) to each chemical structure. Structures without an alert for genotoxicity or without organophosphate/carbamate functionality and evaluated as Cramer class III (high toxicity) were assigned the TTC of 1.5 μg/kg body weight/day. Substances belonging to Cramer class II (intermediate toxicity) were given the TTC of 9 μg/kg body weight/day, whilst the Cramer class I (low toxicity) substances were assigned a TTC of 30 μg/kg body weight/day.

### High-throughput *in vitro* screening

Substances were considered of higher priority in case exposure estimates exceeded the threshold for the respective chemical class or in case substances belong to exclusionary categories of the TTC concept. From the substances that exceeded the TTC, a subset was screened for toxicity alerts in a selection of dedicated prediction models of ToxCast *in vitro* high-throughput screens. For substances where TTC was applicable, the subset of substances with the highest hazard quotient (HQ, ratio exposure/TTC) subjected to screening included 10, 2, and 2 substances for the TTC categories 0.0025, 0.3, and 1.5 μg/kg body weight/day, respectively. In addition, substances from exclusionary categories of the TTC concept with an estimated exposure ≥0.5 μg/kg body weight/day were included. In the context of this study, screening for toxicity alerts involved the use of publicly available results reported for the different toxicity HTS prediction models in the respective publications listed in Table 2, and it should be noted that HTS data were not available for all prioritized substances. Furthermore, the toxicity prediction models were derived from the selection described in a previous manuscript (van der Ven et al., 2020), excluding those without sufficiently accessible underlying numerical data. Although complete coverage of all toxicological endpoints is under development, the applied toxicity prediction models probably represent a wide variety of toxicological domains, at a wide range of biological complexity (from cell functions to even whole organisms); for the purpose of the present case study, these are deemed sufficient to show that prioritized compounds can produce alerts in the proposed strategy. The different toxicity prediction models had different readouts and different approaches to scoring. To enable potency comparison across these different values, the results obtained for the substances of interest were normalized relative to the most potent score per dataset, which was set to 100. The original results for the toxicity prediction models, as well as the normalization calculations and further details on the models are described in Supplementary Table S4.

**TABLE 2:** HTS results for substances with highest Hazard Quotients within each TTC class^a^.

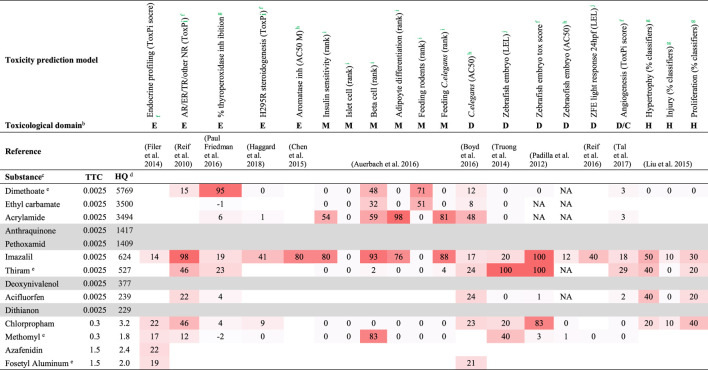

a Highest HQs are the result of highest exposure levels in each TTC category;

b Domains: E, endocrine perturbation; M, metabolic disorder; D, developmental toxicity; C, cancer-related; H, hepatotoxicity;

c Substances are ordered by TTC, then by HQ;

d HQ, Hazard Quotient = ratio Exposure/TTC;

e Dimethoate, thiram, methomyl, and fosetyl aluminum are representative structures in Residue Definitions occurring in the exposure database, of respectively dimethoate (RD, RF-0139-001-PPP), dithiocarbamates (RD, SSD1 RF-0151-001-PPP), methomyl/thiodicarb (RD, SSD1 RF-0293-001-PPP), and fosetyl aluminum (RD, SSD1 RF-0225-001-PPP). The toxicological in vitro screening assays had different readouts, as explained below. To enable potency comparison across these different values, they were normalized relative to the most potent score per dataset, which was set to 100 (note that the original datasets were large, from 309 up to over 10,000 test substances, and that such high potency scores are not necessarily included in the presented selection); logarithmic transformation was applied in case of concentration values (e.g. AC50s). The respective endpoints were

f composed, multiparameter scores (Toxcast’s Toxicological Priority Index, ToxPi), overall endocrine and steroidogenesis scores;

g scaled scores, given as percentage of the maximum score in the test system;

h inhibitory or activating concentrations (AC50s);

i rank in an ordered potency list; or

j lowest effect level (LEL) in a dose range. NA, not active. Anthraquinone, pethoxamid, and deoxynivalenol were not included in either of the test sets. Original results, normalization calculations, and further details of the screening models are available in Supplementary Table S4. Increasing color shading reflects increasing relative potency, supporting the numerical values for the screening models.

## Results

### Substances with exposure data

**FIGURE 1:**
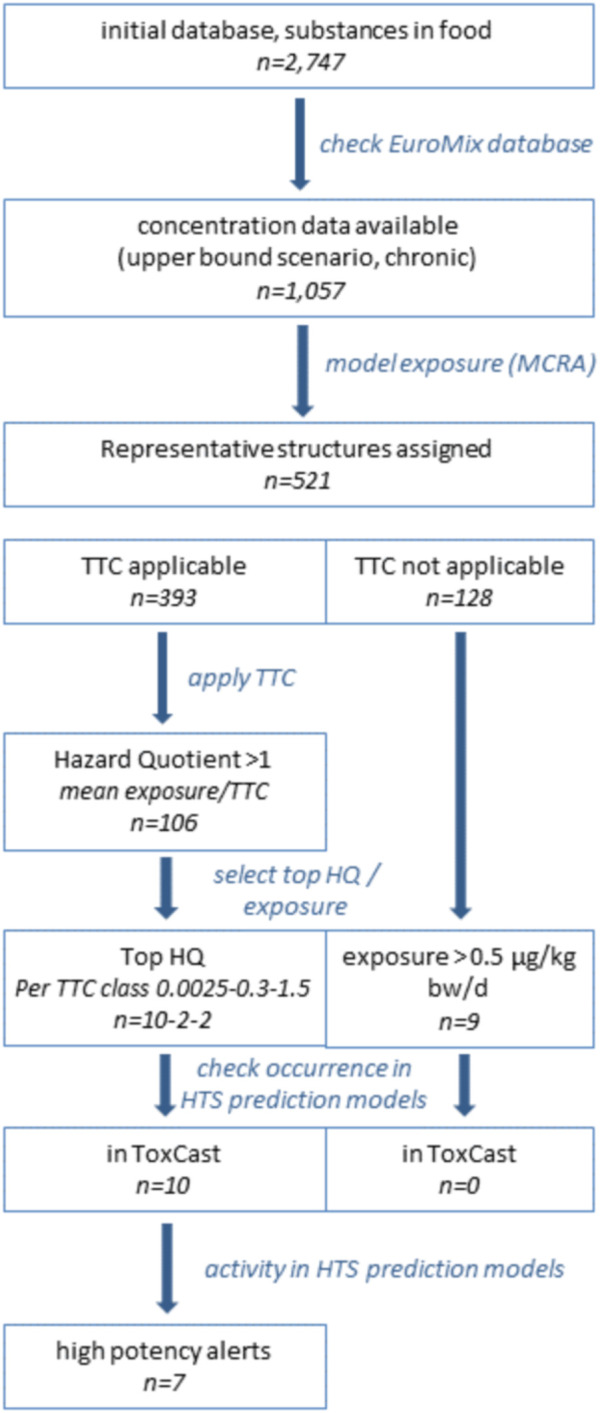
Flow of consecutive steps in the next generation risk assessment case study.

The first step in the exposure-driven selection of chemicals was the identification of chemical substances that may occur in food. Focusing on chemicals in food for which data can be submitted to EFSA resulted in a list of over 2,700 substances. From these, only those with available analytical data were selected for the case study, because calculating dietary exposure estimates requires concentration data in food. Using the EuroMix database with concentration data, we identified 1,057 candidates for inclusion in the case study (Figure 1). It should be noted that these are not all unique chemicals, since the list also comprised groups of chemicals, such as ‘dioxins and dioxin-like PCBs’, or residues of pesticides. The residue definition of pesticides, given as “RD” (residue definition) in the database, represents the sum of analyzed parent compounds and metabolites. The toxicological evaluation (TTC and HTS using prediction models) was done for the parent compound as representative for the respective RD group. Regarding pesticides, this may lead to a worst case high hazard quotient, when considering that metabolites generally are less active in comparison to their parent compound. Dietary exposures estimates for the substances of interest were calculated using the Monte Carlo Risk Assessment tool (see Methods). The resulting estimates for dietary exposure in a chronic scenario (upper bound) are listed in Supplementary Table S1. Not surprisingly, nitrate, a chemical that is found in many foods at relatively high levels, especially in green leafy vegetables, was the chemical with the highest daily intake.

### Threshold of toxicological concern

Representative chemical structures were assigned to the 1,057 entries for which an upper bound exposure concentration was calculated (Figure 1). To this end the coupling between Substance identity code (SSD1) and CAS-registry number information as present in the EuroMix Inventory database was used. This yielded a chemical structure for 521 entries (of the 1,057; Figure 1). Completing the set of chemical representative structures for all 1,057 would definitely be possible, but was not deemed necessary for our purpose (illustration of the prioritization concepts). Substances that were not evaluated in this exercise (due to absence of a representative structure, Supplementary Table S2, tab “TTC evaluation”) are not to be considered as safe; they can be considered awaiting further prioritization.

Application of the TTC concept was not possible for 128 of the set of 521 entries, following the standard exclusion rules (Figure 1). These substances were maintained in the case study (Supplementary Table S3, second worksheet), but no safe threshold for exposure can be assigned to these entries. For the remaining 393 substances, a TTC was derived and compared to the estimated levels of exposure in an upper bound scenario (Supplementary Tables S2, S3). In total, 106 of the 393 substances with a TTC showed estimated exposures exceeding the threshold for a low probability of adverse health effect (Supplementary Table S3, first worksheet). Of these 106 substances with a HQ > 1, the majority (95 substances) was assigned the lowest TTC based on the presence of a structural alert for genotoxicity.

### High-throughput *in vitro* screening

Using TTC as a pragmatic filter to identify substances of higher priority yielded two lists: one for which TTC was applied (exposure exceeding TTC), and one for which the TTC concept is not applicable (Figure 1; Supplementary Table S3). The prioritized substances were screened for their toxicological potential using a selection of prediction models based on HTS assays, to illustrate how follow-up assessment could work out. In this illustrative assessment, the screening for toxicological alerts was limited to substances with the highest HQ from the three represented TTC classes in the first list (TTC applicable): 10, 2, and 2 substances for the TTC categories 0.0025, 0.3, and 1.5 μg/kg body weight/day, respectively (Figure 1; Table 2). These substances, for which the exposure estimates exceeded the TTC (HQ > 1), variably overlapped with substances included in reports for toxicity prediction models, and no HTS data were even available for anthraquinone, pethoxamid, deoxynivalenol, and dithianon (Table 2). The variable count of alerts per substance in Table 2 thus at least in part represents lack of inclusion in a HTS toxicity prediction model, and not a true absence of toxicological potential per se. In addition, seven substances from the second list (TTC not applicable), having a mean exposure above the arbitrary limit of 0.5 μg/kg body weight/day were explored. However, none of these was reported in any of the toxicity prediction models studied. This means that information on a possible toxicity alert from any of the seven substances was lacking.

The selected HTS toxicity prediction models covered a variety of toxicological domains, including developmental toxicity, hepatotoxicity, endocrine perturbation and metabolic disorder. Various screening paradigms were applied in the selected models; however, most models used compiled scores to represent the results of multiple assays (up to several hundreds, see Supplementary Table S4) relevant to the studied endpoint. One such a well-established multiparameter score in this context is the Toxicological Priority Index (ToxPi) (Reif et al., 2010). Other models, notably the zebrafish embryo model, used multiple classifiers in a single test system (Padilla et al., 2012). Results were also reported in different ways, either as ToxPi score, percentage activity, rank, or active concentration (lowest or 50% active concentration (AC50); Supplementary Table S4). In Table 2, these different scores are represented in a normalized way, to enable potency comparison across the models (normalization calculations are given in Supplementary Table S4).

To highlight some key observations, it can be concluded that of the 10 substances for which results were reported from the prediction models, seven substances (i.e., dimethoate, ethyl carbamate, acrylamide, imazalil, thiram, chlorpropham, and methomyl) appeared to have at least one major alert for a toxicological effect and one substance (acifluorfen) showed moderate potency (Table 2). The relatively modest activity of the remaining two substances, i.e., azafenidin and fosetyl aluminum (Table 2) may be due to the fact that these two substances were hardly represented in the set of prediction models used here. A notably active compound in the included models was imazalil, which, in contrast to another well represented substance, i.e., thiram, induced strong alerts in the metabolic disorder domain. An illustrative example of variable overlap among models is in developmental toxicity, where only slight or moderate potency alerts were observed with eight substances in the *C. elegans* model, whereas three of these substances showed a strong alert in either or both of the zebrafish embryo models.

## Discussion

There is a growing demand for more hazard information on an ever increasing number of chemical substances, e.g., in chemical product development or in databases of identified food contaminants. A rational approach to resolve this challenge would be to reduce the number of chemicals that would require risk assessment, e.g., through “chemical simplification” as proposed recently (Fenner and Scheringer 2021) and to aim for a circular economy (Kümmerer, Clark, and Zuin 2020). However, the high impact of such a paradigm shift would imply a long term for implementation, and therefore short-term, pragmatic strategies to alleviate the pressure on risk assessment resources are needed. One such a strategy is to prioritize substances for risk assessment, where prioritization is a step that should precede the actual risk assessment, without intending to waive risk assessment. Prioritization should then lead to a focus on the highest risk, spending resources on those substances where the highest gains (in terms of risk) can be realized. Therefore, the goal of the present case study was to explore how exposure estimates can be combined with initial hazard estimation (through the TTC), to identify chemicals that are of higher priority to undergo a more extensive toxicological evaluation. The latter should preferably be done using NAMs, but not excluding more traditional toxicological methods. To test our approach, we choose to focus on chemicals in food. By making use of the EuroMix database as a data source, the case study was limited to oral exposure, to defined groups of chemicals (i.e., plant protection products, their residues and environmental contaminants) and to a defined region (i.e., Europe). Furthermore, the exposure estimates in the case study were limited to the Dutch population aged 7–69 years. A further caveat to the final selection is inherent to the applied prioritization criteria, as for instance the set of substances excluded by the exposure criterion may still contain (high-potency) toxicants, and application of TTC could have been expanded by assigning representative chemical structure information to more substances. However, while considering these limitations, the purpose of this study, that is to demonstrate proof-of-principle for the methodology, can still be met.

The first step towards priority-setting is to estimate dietary exposure levels. Here, exposure was estimated with intermediate tier modelling: using exposure distributions based on individual food consumption data combined with measured concentration data in food. In principle, lower tiers using population statistics such as PRIMO or GEMS data sheets together with maximum (residue) levels could also serve as input, although these models are not fit for high-throughput screening of large numbers of substances. Since the aim of the case study was prioritization and not an actual risk assessment, databases were not scrutinized for completeness. Therefore, we did not check whether the EuroMix database contains concentration data for all relevant foods in which a certain chemical is expected to be present at a sufficient sample size. We also did not use processing factors for all substances and agricultural use data for pesticides. This could be done in a refinement step for prioritized chemicals. Furthermore, exposure estimates for all substances should ideally be generated using automated screening of a concentration database such as implemented in the high-throughput version of the U.S. EPA Stochastic Human Exposure and Dose Simulation Model (SHEDS-HT; (Isaacs et al., 2014)). For MCRA, this feature has not been implemented yet. Therefore, we used the cumulative exposure assessment tool of MCRA as a work-around for a screening tool, which may have caused deviations of the single substance exposure percentiles. However, the most recent version of MCRA (version 9.1; https://mcra.rivm.nl, accessed July 2022) contains a prioritization tool, which allows screening of multiple single chemical exposure assessments within a single run. This greatly facilitates prioritization of chemicals.

The next step to identify substances of higher priority is application of a hazard estimate, for which we used the TTC, to derive a hazard quotient. The TTC estimates toxicological thresholds for a wide range of substances based on their chemical structures, and can be used to assess the likelihood that a particular level of exposure to a new chemical would be of low toxicological concern (European Food Safety Authority, 2019b). The most conservative threshold (0.0025 μg/kg body weight/day) is assigned to substances that may be DNA-reactive mutagens. Here, we applied modules available in the OECD QSAR Toolbox and in Derek Nexus to screen for genotoxic potential, covering DNA reactivity as well as other genotoxic modes of action. Considering also *in silico* alerts for chromosome aberration - and not only for Ames mutagenicity—is likely to be the reason for the relatively high number of substances with TTC for mutagenicity assigned (∼25%). It is a conservative approach, since the cancer TTC value is derived from carcinogenicity studies of substances that are likely to be carcinogenic by DNA-reactivity (Boobis et al., 2017). The very low threshold (0.0025 μg/kg body weight/day) that is extrapolated from these studies to reflect the very low acceptable risk on additional tumors for a life-time exposure (1:1.000.000) is likely to be protective for substances with other genotoxic modes of action. A study on the level of protection that is (implicitly) accepted for non-genotoxic carcinogens by not having a separate threshold for non-genotoxic carcinogens shows that the higher TTC values (derived for the groups of organophosphates and the three Cramer Classes) have to be associated with a potentially much higher risk on additional tumors for a life-time exposure due to non-genotoxic carcinogenicity mechanisms (∼1:1,000) (Braakhuis et al., 2018). Furthermore, the use of a battery of *in silico* alert models, covering both DNA reactivity and other genotoxic modes of action, increases the probability that an alert is identified, and thus increases the number of false positive identifications of genotoxicity. True genotoxicity of the as such labeled substances in our work flow has not been evaluated. However, as most of these are plant protection products approved for EU market entry, they are not likely to be true genotoxicants, and a higher TTC would be applicable, lowering their hazard quotient. In that case, exposure of only 19 of the 87 labeled genotoxicants would exceed the next lowest TTC level (0.3 μg/kg body weight/day, applicable to organophosphates and carbamates), and exposure of another five remaining substances would exceed the TTC level associated with Cramer Class III (high toxicity; 1.5 μg/kg body weight/day). Hence, 11 of the 17 substances identified as priority substances in Table 2 may be of less concern than assumed in this work flow. The most efficient way to resolve this issue for the present case study would be to collect existing experimental data from appropriate *in vitro* genotoxicity tests, including mutagenicity tests in bacteria and mammalian cells, for the substances with genotoxicity alerts in the applied *in silico* models. Future application of the proposed prioritization strategy would benefit from a weight of evidence approach combining *in silico* models for DNA-reactivity with appropriate *in vitro* mutagenicity assays. Review of the cancer potency database underlying the cancer TTC value, as proposed by Boobis and co-workers (Boobis et al., 2017), supports the application of Cramer class thresholds to all non-genotoxic compounds, including non-genotoxic carcinogens (Batke et al., 2021).

Overall, the current TTC concept has limited applicability, because it is not substance specific. The TTC concept does not inform on the toxicological potential of a substance of interest, but only provides risk thresholds for classes of structurally similar substances. Furthermore, it reflects the risk for only those apical endpoints that were included in the toxicity studies used to derive the threshold values (the Munro database of toxicity studies; (Munro et al., 1996)). A critique on the TTC concept is that systemic effects currently of high concern, like neurotoxicity, immunotoxicity, and endocrine disruption, were not included when deriving the thresholds. The same applies to more acute effects like allergies, hypersensitivity or intolerances; these are also not covered by the approach (Bschir 2017). Although the thresholds are based on a distribution of toxicities EFSA expected that other apical endpoints (with the exception of endocrine disruption) will not shift the distribution of NOAEL (No-Observed-Adverse-Effect-Level) values to lower values (European Food Safety Authority, 2019b)) and references therein). The lack of information on various health effects, including epigenetic multigeneration and teratogenic effects, adds to the uncertainty of the TTC concept, but this applies equally to the risk assessment of the majority of substances for which no multigeneration or complete developmental toxicity study is available. Another source of uncertainty is the lack of robust knowledge about the mechanisms of low-dose or mixture effects and non-monotonic dose-response relationships (Bschir 2017). Overall, it can be concluded that the TTC system could be refined, for which input is required from more recent chronic toxicity data from a larger database of chemicals, with more diverse structures, such as available in industry.

To illustrate how toxicological potential could be established in a follow-up assessment, the final subset of prioritized substances was screened for toxicological potential using a selection of HTS toxicity prediction models. Comparison among these substances, *e.g.* regarding potency or specificity, or association of TTC level with produced results, is challenging because of uneven inclusion of substances across HTS studies. A more comprehensive toxicological evaluation, including a larger number of HTS prediction models, as well as continued testing of substances, may improve this picture. Still, it appears that some distinction can be made among substances that are well-represented: e.g., imazalil is active in a broad, non-specific manner, in contrast to thiram, which showed mainly potent effects for development, or to acrylamide, which may have more specific effects in the metabolic disorder domain. Regarding the HTS toxicity prediction models, it seems that parallel approaches in a given domain may produce variable results. This is for instance observed for overall endocrine activity revealing no active compounds in one prediction model (Filer et al., 2014) and several hits in another model (Reif et al., 2010). Similarly, various assays in zebrafish embryos produced variable results among substances (Padilla et al., 2012; Truong et al., 2014; Reif et al., 2016), indicating that the choice of the assessed endpoint (in the same model) affects the distribution of relative potency of the substances. It is therefore imperative to develop multiple prediction models per toxicological domain, and to evaluate each of these models for scientific confidence (Cox et al., 2014; Patlewicz et al., 2015). Overall, it should be emphasized that in this case study the retrieved alerts for toxicological potential are merely illustrative, because only a limited number of HTS-based prediction models were available and/or screened for activities of the prioritized substances. A more comprehensive screening, including toxicity prediction models based on both HTS assays and *in silico* approaches, could produce more refined (priority) alerts. These results could also be used as guidance for more complex follow-up assays to confirm and quantify specific effects.

In the applied methodology, exposure was assessed for chemicals for which analytical data were available. However, for new chemicals entering the market those data will not be present. By making use of field trial data as input for exposure modeling, this approach would also be applicable for new pesticides entering the market. Alternatively, models for predicting concentrations levels in food and other relevant exposure sources such as air and dust, e.g., based on chemical and use similarities, would be needed. In this way, prioritization can be supportive to a next generation risk assessment strategy, because it would provide guidance for selection of substances for further toxicological screening, through NAMs in the first place, but not excluding other methods for toxicity testing. Of course, alternative approaches such as *a priori* HTS for toxicological potential remain valid, and substances for which hazard alerts are thus detected are already prioritized for follow-up assessment in that way. However, the observation that no HTS data were available for four of the substances that were prioritized by our paradigm underlines the importance of an approach for prioritization that does not build on toxicological screening in the first place.

On the other hand, priority alerts were detected in a selection of substances that are already on the market and represent relevant exposure in consumers. The approach may therefore not only be applied to new substances, but also for prioritizing existing substances for re-evaluation, such as substances emerging from suspect screening analyses of human biomonitoring samples for which the toxicological dossier appears to be limited, or chemicals to be grouped in assessment groups for mixture risk assessment.

In this case study we applied a prioritization approach, focusing on exposure to substances that have been registered or are known to occur in food, such as food contact materials (Van Bossuyt et al., 2017). The purpose was to explain the possibilities and uncertainties of the employed approach, not to present an all-inclusive prioritization or a comprehensive toxicological evaluation of prioritized substances. Evidently, the number of missed priority substances will be higher with more and more restrictive selection criteria. More inclusive variations of the employed approach could be made through omitting any exposure bound, or even screen the entire initial database in HTS prediction models. A rationale for selection criteria should follow from a dedicated problem formulation, and the presented approach could help to define such criteria.

Overall, the method of prioritization of chemicals applied in this case study has the potential to contribute in a new strategy for risk assessment, considering that the final evaluation requires an uncertainty analysis regarding comprehensiveness of applied models, and regarding addressing the original assessment question.

## Data Availability

The original contributions presented in the study are included in the article/Supplementary Materials, further inquiries can be directed to the corresponding author.
